# Effects of fish oil supplementation on inflammatory markers in chronic heart failure: a meta-analysis of randomized controlled trials

**DOI:** 10.1186/1471-2261-12-77

**Published:** 2012-09-20

**Authors:** Wei Xin, Wei Wei, Xiaoying Li

**Affiliations:** 1 First Department of Geriatric Cardiology, Chinese PLA General Hospital, Beijing, Peoples Republic of China; 2 Department of pathophysiology, Medical College of Nankai University, Tianjin, Peoples Republic of China

**Keywords:** Fish oil, Omega 3 polyunsaturated fatty acids, Heart failure, Inflammatory markers, Meta-analysis

## Abstract

**Background:**

Effects of fish oil on systematic inflammation in chronic heart failure remain unclear. In this meta-analysis, we aimed to evaluate the influence of fish oil supplementation on circulating levels of inflammatory markers in patients with chronic heart failure.

**Methods:**

Human randomized controlled trials, which compared the effects of fish oil supplementation with placebo in patients with chronic heart failure, were identified by systematic search of Medline, Embase, Cochrane’s library and references cited in related reviews and studies up to November 2011. Outcome measures comprised the changes of circulating inflammatory markers. Meta-analysis was performed with the fixed-effect model or random-effect model according to the heterogeneity.

**Results:**

A total of seven trials with eight study arms were included. The pooled results indicated circulating levels of tumor necrosis factor α (SMD = -0.62, 95% CI -1.08 to -0.16, p = 0.009), interleukin 1 (SMD = -1.24, 95% CI -1.56 to -0.91, p < 0.001) and interleukin 6 (SMD = -0.81, 95% CI -1.48 to -0.14, p = 0.02) were significantly decreased after fish oil supplementation; however, high sensitivity C reactive protein, soluble intracellular adhesion molecular 1 and vascular cell adhesion molecular 1 were not significantly affected. Meta-regression and subgroup analysis results suggested the difference in dose of fish oil and follow-up duration might influence the effects of fish oil on tumor necrosis factor α and interleukin 6. Greater reduction of these two markers might be achieved in patients taking fish oil of a higher dose (over 1000 mg/day) or for a longer duration (over 4 months).

**Conclusions:**

Limited evidence suggests anti-inflammation may be a potential mechanism underlying the beneficial effects of fish oil for chronic heart failure. Further large-scale and adequately powered clinical trials are needed to confirm these effects.

## Background

Chronic heart failure (CHF) is a common clinical syndrome which can be caused by various cardiovascular disorders [1-3]. Despite recent advances in therapeutic strategies, CHF is still one of the leading causes of morbidity and mortality worldwide [4,5]. Although current treatments improve clinical symptoms and slow progression of cardiac dysfunction, the prognoses for patients with CHF remain poor [5,6]. Therefore, there is an urgent need for development of novel therapy for CHF.

Fish oil, mainly consisting of two categories of marine omega 3 polyunsaturated fatty acids (PUFAs) - eicosapentaenoic acid (EPA) and ducosahexanoic acid (DHA), has been suggested to be a potential adjunctive therapy for many cardiovascular disorders, including CHF [7,8]. A recent clinical trial [9] showed a statistically significant improved mortality or cardiovascular hospitalizations (-8%; p < 0.01) in patients with CHF who received additional fish oil supplementation. However, the exact mechanisms underlying benefits of fish oil to patients with CHF are not fully understood. Inflammation plays an important role in the pathogenesis and progression of CHF [10,11]. Levels of circulating inflammatory markers, including high sensitivity C reactive protein (hsCRP), tumor necrosis factor α (TNF-α), interleukin 1 (IL-1), interleukin 6 (IL-6), soluble intracellular adhesion molecule-1 (sICAM-1) and soluble vascular cell adhesion molecule-1 (sVCAM-1) et al., were elevated in patients with CHF, serving as prognostic markers or therapeutic targets [11-14]. Fish oil has been suggested to exert anti-inflammation effects in patients with CHF [7,8,15]. However, clinical trials in humans have shown inconsistent results [16-23]. Herein, we aimed to evaluate the effects of fish oil on levels of the above inflammatory markers in CHF through meta-analysis of randomized, placebo-controlled clinical trials.

## Methods

### Search strategy

We systematically searched Pubmed (from 1950 to November, 2011), Embase (from 1966 to November, 2011) and the Cochrane Library (Cochrane Center Register of Controlled Trials) for relevant records, using the term “omega-3 fatty acids”, “fish oil”, “fish-oil”, “marine oil”, “eicosapentaenoic acid”, “EPA”, “docosahexaenoic acid”, “DHA”, “dietary therapy” paired with the following: “heart failure”, “cardiac failure”, “cardiac dysfunction”, “ventricular dysfunction”, “ventricular insufficiency”, and “cardiomyopathy”. The search was limited to studies in human. We also analyzed reference lists of original and review articles using a manual approach.

### Study selection

Studies were selected for analysis if they met the following criteria: 1) published as full-length articles in any language; 2) reported as a prospective, randomized, and placebo-controlled trial with either a parallel or a crossover design (regardless of sample size); 3) analyzed adult patients with established CHF (regardless of the etiology and severity of the disease) who were assigned to oral fish oil supplementation or placebo for at least one month in addition to concurrent therapy; 4) reported data on levels of at least one of the following circulating inflammatory factors, including hsCRP, TNF-α, IL-6, IL-1, sICAM-1 or sVCAM-1.

### Data extraction and quality assessment

Searching, data extraction, and quality assessment were completed independently by two authors (WX and WW) according to inclusion criteria. Discrepancies were resolved by consensus. Extracted data included study design characteristics, patient characteristics (number, age, sex, cardiac function, baseline circulating levels of inflammatory markers, and concurrent medications), intervention strategies (dose of EPA and DHA, ratio of EPA to DHA and composition of placebo), follow-up duration, and means and standard deviations (SDs) for changes of the aforementioned inflammatory markers from baseline. If the study provided medians and interquartile ranges (IQRs) instead of means and SDs, we imputed the means and SDs as described previously [24]. For studies with multiple intervention groups (e.g. with different doses of fish oil), we split the shared control group into two or more groups with smaller sample size to overcoming a unit-of-analysis error, and included these two or more comparisons into the meta-analysis according to the instruction of Cochrane’s Handbook [25].

The quality of the studies was judged by quality of randomization, generation of random numbers, concealment of treatment allocation, blinding, and reporting of withdrawals. Trials scored one point for each area addressed, with a possible score between 0 and 5, where 5 represented the highest level of quality [26].

### Statistical analysis

All endpoints were estimated based on the change from baseline to follow-up, and pooled effects were presented as standardized mean difference (SMD) with 95% confidence intervals (CI) because various measurements were applied in the included studies. Inter-study heterogeneity was formally tested using Cochrane’s Q test, and significant heterogeneity was considered existing if p value was < 0.10. The I^2^ statistic was also examined, and a value of I^2^ > 50% indicated significant heterogeneity among the trials [27]. If above tests showed no significance of heterogeneity among the included trials, fixed-effect model was used to calculate SMD and its 95% CI. By contrast, if there is significant heterogeneity among the included studies, random-effect model was applied [28]. Meta-regression analysis and predefined subgroup analysis was performed to explore the possible source of heterogeneity. Furthermore, potential publication bias was assessed with Egger regression asymmetry test [29] and funnel plots; p values were two-tailed and statistical significance was set at 0.05. Meta-analysis and statistical analysis was performed with Stata software (version 12.0; Stata Corporation, College Station, TX) and RevMan software (version 5.1; Cochrane Collaboration, Oxford, United Kingdom).

## Results

### Search results

**Figure 1:**
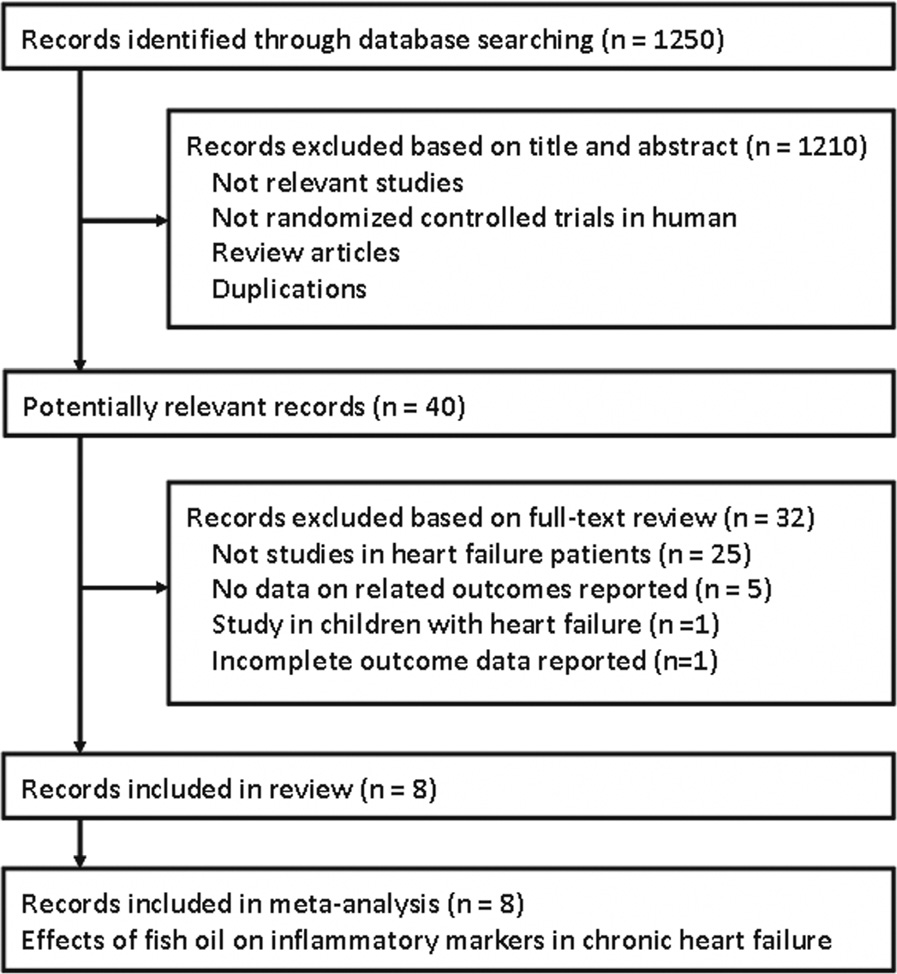
Flow diagram of the study selection procedure.

A total of 1250 records were identified, and 1210 were excluded because they did not describe randomization or controlling, or because the objectives of these studies were irrelevant to the present meta-analysis. Of the 40 potentially relevant records screened, eight met the selection criteria for the current meta-analysis [16-23] (Figure 1). Thirty-two records were excluded because the participants in 25 trials were not patients with CHF; 5 trials didn’t report any data of related outcomes; 1 trial included children with CHF and 1 trial reported incomplete data of related outcomes. 

### Study characteristics

Two of the included records reported the outcomes of different inflammatory markers from the same study population (one record of hsCRP [21], the other of TNF-α and IL-6 [22]), which makes a total of 7 trials included. This trial by Moertl et al. [21,22] included two intervention groups of different doses of fish oil (a higher dose group of 3360 mg/d and a lower dose group of 840 mg/d), which we included in our study separately. Overall, a total of 8 study arms from 7 trials were included in this meta-analysis (Table 1 and Table 2), of which 6 had a parallel design [16,18-23] and 1 had a crossover design [17]. 

**Table 1:** Overview and characteristics of included studies

**Study**	**No.of patients**	**Mean age**	**Male**	**Ischemic etiology**	**Mean NYHA**	**Mean LVEF**	**ACEI/ARB**	**β-blocker**	**Statins**	**EPA + DHA dose**	**EPA dose**	**DHA dose**	**EPA/DHA**	**Duration**	**Placebo**	**Study design**	**Quality score**
		**y**	**%**	**%**		**%**	**%**	**%**	**%**	**mg/d**	**mg/d**	**mg/d**		**months**			
**Mehra 2006**	14	60	71	43	III-IV	17	93	64	0	5440	3520	1920	1.83	4	Corn oil	R, DB, PC	3
**O'Keefe 2006**	18	68	100	100	NR	NR	NR	78	83	810	225	585	0.38	4	Corn oil & olive oil	R, DB, PC, CO	3
**Nodari 2009**	44	63	91	0	II-III	36.2	100	100	NR	1440	540	900	0.60	6	Olive oil	R, DB, PC	3
**Zhao 2009**	75	73	73	64	II-III	31	100	87	NR	600	360	240	1.50	3	NR	R, SB, PC	3
**Eschen 2010**	138	60	86	68	II-III	32	95	88	54	900	490	410	1.20	6	Olive oil	R, DB, PC	3
**Moertl 2011 a**	22	57	83	0	III-IV	24.3	100	100	NR	840	465	375	1.24	3	Gelatin	R, DB, PC	5
**Moertl 2011 b**	21	60	94	0	III-IV	24.2	100	100	NR	3360	1860	1500	1.24	3	Gelatin	R, DB, PC	5
**Nodari 2011**	133	63	75	0	I-II	36.5	100	100	14	1950	730	1220	0.60	12	Olive oil	R, DB, PC	3

The study by Moertl et al. includes two intervention groups of different dosages of EPA + DHA (Moertl 2011a, EPA + DHA 840 mg/day and Moertl 2011b, EPA + DHA 3360 mg/day).

NYHA, New York Heart Association; LVEF, left ventricular ejection fraction; ACEI, angiotensin-converting enzyme inhibitor; ARB, angiotensin receptor blocker; EPA, eicosapentaenoic acid; DHA, ducosahexenoic acid; R, random; DB, double blind; SB, single blind; PC, placebo-controlled; CO, crossover; NR, not reported.

**Table 2:** Mean baseline circulating levels of the inflammatory markers

**Study**	**hsCRP**	**TNF-α**	**IL-1**	**IL-6**	**sICAM-1**	**sVCAM-1**
	**μg/ml**	**pg/ml**	**pg/ml**	**pg/ml**	**pg/ml**	**pg/ml**
**Mehra 2006**	NR	1.5	NR	NR	NR	NR
**O'Keefe 2006**	1.3	6.7	NR	1.6	NR	NR
**Nodari 2009**	NR	19.0	402.0	7.15	NR	NR
**Zhao 2009**	5.6	61.5	NR	11.3	1.0	1.6
**Eschen 2010**	2.1	NR	NR	NR	0.3	0.7
**Moertl 2011 a**	5.0	2.5	NR	3.7	NR	NR
**Moertl 2011 b**	5.0	2.3	NR	3.2	NR	NR
**Nodari 2011**	NR	23.0	483.0	10.6	NR	NR

The study by Moertl et al. includes two intervention groups of different dosages of EPA + DHA (Moertl 2011a, EPA + DHA 840 mg/day and Moertl 2011b, EPA + DHA 3360 mg/day).

hsCRP, high sensitivity C reactive protein; TNF-α, tumor necrosis factor α; IL-1, interleukin 1; IL-6, interleukin 6; sICAM-1, soluble intracellular adhesion molecule-1; sVCAM-1, soluble vascular cell adhesion molecule-1; NR, not reported.

All of the studies declared that the participants were clinically and hemodynamically stable CHF patients who received optimal medical therapy based on modern CHF treatment strategies, including diuretics and neurohormonal inhibitors, and the medication treatment was maintained during the follow-up interval. Statins were also taken by 83%, 54% and 14% of the participants in three of the studies [17,20,23], not taken in one study [16], and not reported in the rest three studies [18,19,21,22]. The mean age of the participants of the included studies varied from 57 to 73. The etiology of CHF is exclusively ischemic in 1 trial [17], non-ischemic in 3 trials [18,21-23], and from both in 3 trials [16,19,20]. Only one study reported inclusion of the CHF patients primarily caused by myocarditis (5 of the 43 included participants) [22]. However, patients with acute myocarditis were excluded from this study. The baseline cardiac functions were of classes I – IV defined by New York Heart Association (NYHA) and the baseline left ventricular ejection fraction (LVEF) of the participants varied from 17% to 36%. The dosage of fish oil (calculated as total dose of EPA and DHA) ranged from 600 mg/day to 5440 mg/day, with the ratio of EPA to DHA varying from 0.38 to 1.83. The duration of the investigation varied from 3 months to 12 months.

### Data quality

The quality score of the 7 trials ranged form 3 to 5. All of the included trials were randomized and placebo-controlled, with 6 studies in a double-blind fashion [16-18,20-23] and 1 in a single-blind fashion [19]. Two of the trials reported methods of random sequence generation [19,21,22], while only one study reported allocation concealment [21,22]. Details of withdrawals were reported in all of the 7 included trials.

### Effects of fish oil supplementation on hsCRP in CHF

**Figure 2:**
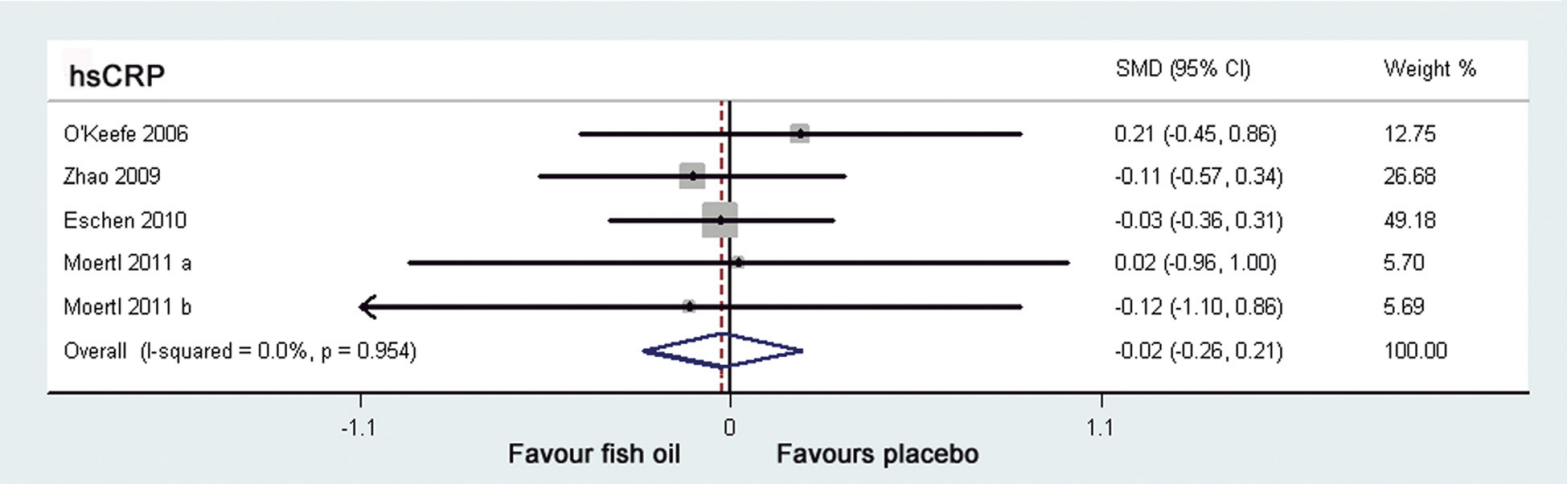
**Forest plot from meta-analysis of standardized mean difference in circulating high sensitivity C-reactive protein for patients with CHF randomized to fish oil or placebo.** The effect size of each study is proportional to the statistical weight. The diamond indicates the overall summary estimate for the analysis; the width of the diamond represents the 95% CI. CHF, chronic heart failure; hsCRP, high sensitivity C reactive protein; SMD, standardized mean difference; CI, confidence interval.

Four trials [17,19-21] including 5 study arms investigated the effects of fish oil supplementation on hsCRP levels in CHF patients (Table 2), but none of these studies showed a significant effect. No significant heterogeneity was found between the 5 study arms (I^2^ = 0.0%, p = 0.954), therefore the fixed-effect model was applied. The pooled results indicated that additional supplementation of fish oil didn’t significantly reduced the circulating level of hsCRP (SMD = -0.02, 95% CI -0.26 to 0.21, p = 0.84; Figure 2) in patients with CHF. Sensitivity analysis, excluding the two study arms in which hsCRP data were imputed from median and IQRs [21], also suggested an insignificant effect (SMD = -0.02, 95% CI -0.27 to 0.23, p = 0.87). 

### Effects of fish oil supplementation on TNF-Î±

**Figure 3:**
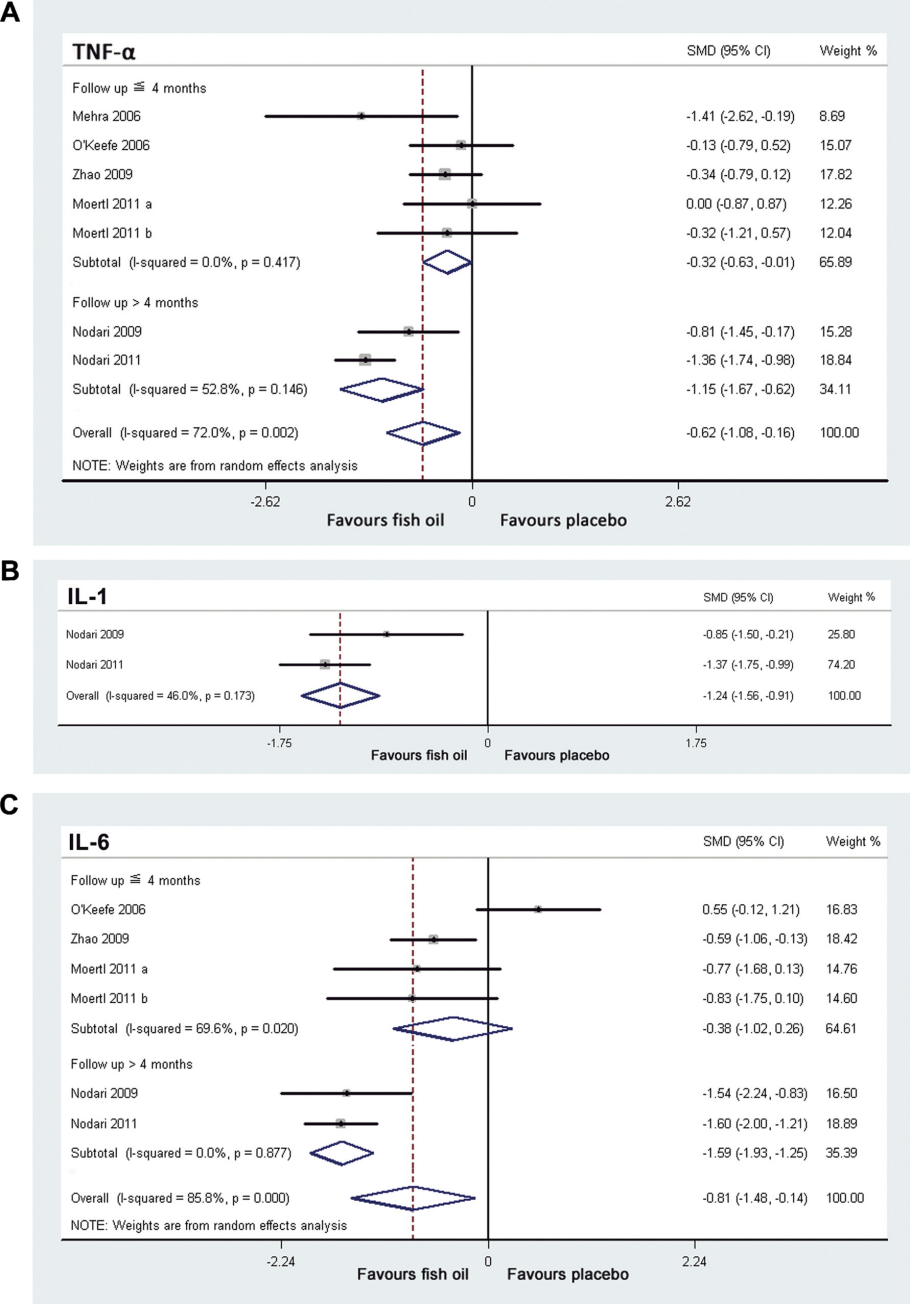
**Forest plots from meta-analysis of standardized mean difference in circulating tumor necrosis factor α (A), interleukin 1 (B) and interleukin 6 (C) for patients with CHF randomized to fish oil or placebo.** The effect size of each study is proportional to the statistical weight. The diamond indicates the overall summary estimate for the analysis; the width of the diamond represents the 95% CI. CHF, chronic heart failure; TNF-α, tumor necrosis factor α; IL-1, interleukin 1; IL-6, interleukin 6; SMD, standardized mean difference; CI, confidence interval.

Six trials [16-19,22,23] including 7 study arms reported the effects of fish oil supplementation on TNF-α in CHF (Table 2). Significant heterogeneity was found among these 7 study arms (I^2^ =72.0%, p = 0.002). The pooled analysis of the overall effect suggested that fish oil significantly reduced the level of circulating TNF-α (SMD = -0.62, 95% CI -1.08 to -0.16, p = 0.009; Figure 3A) in patients with CHF. Sensitivity analysis, excluding the two study arms in which TNF-α data were imputed from median and IQRs [22], didn’t affect the result (SMD = -0.77, 95% CI -1.32 to -0.22, p = 0.006). 

In view of the fact that statistical heterogeneity existed across the enrolled study arms, we performed meta-regression analysis including some predefined covariates to explore the potential sources of heterogeneity. The results indicated that follow-up duration was negatively related to effect size (regression coefficient = -0.12, 95% CI -0.19 to -0.05, p = 0.009), which largely explained the heterogeneity of the effect (Figure 3A). The mean age, gender of the participants, etiology of CHF, baseline LVEF, baseline TNF-α, doses of EPA or DHA, total dose of fish oil and the ratio of EPA to DHA were not significant modifiers to the effects of fish oil supplementation on TNF-α.

Additionally, we conducted a predefined subgroup analysis to observe the influence of study characteristics to the effects of fish oil supplementation on TNF-α (Table 3). The results showed that the differences in total dose of fish oil and the follow-up duration might influence the effects of fish oil on TNF-α significantly. Specifically, fish oil supplementation with total dose over 1000 mg/day or for duration over 4 months seemed to associate with a more remarkable reduction of TNF-α.

**Table 3:** Subgroup estimation of the effects of fish oil supplementation on TNF-α and IL-6 in patients with CHF

	**TNF-α**			**IL-6**		
**Subgroup**	**Study arms (n)/patients (n)**	**SMD [95% CI]**	**p**^**※**^	**Study arms (n)/patients (n)**	**SMD (95% CI)**	**p**^**※**^
**Age**						
≤ 60 years	3/57	-0.47 [-1.22, 0.27]		2/43	-0.80 [-1.45, -0.15]	
> 60 years	4/285	-0.68 [-1.28, -0.08]	0.67	4/285	-0.81 [-1.72, 0.10]	0.99
**Gender (male)**						
≤ 90%	4/244	-0.76 [-1.48, -0.03]		3/230	-1.02 [-1.77, -0.28]	
> 90%	3/98	-0.44 [-0.87, -0.02]	0.46	3/98	-0.60 [-1.92, 0.72]	0.58
**Ischemic CHF**						
Not excluded	3/125	-0.42 [-0.94, 0.09]		2/111	-0.05 [-1.17, 1.07]	
Excluded	4/217	-0.71 [-1.34, -0.07]	0.50	4/217	-1.33 [-1.74, -0.92]	0.06
**Baseline NYHA (class IV)**						
Not included	3/249	-0.85 [-1.51, -0.18]		3/249	-1.23 [-1.93, -0.54]	
Included	3/57	-0.47 [-1.22, 0.27]	0.47	2/43	-0.80 [-1.45, -0.15]	0.37
**Baseline LVEF**						
≤ 30%	3/57	-0.47 [-1.22, 0.27]		2/43	-0.80 [-1.45, -0.15]	
> 30%	3/249	-0.85 [-1.51, -0.18]	0.47	3/249	-1.23 [-1.93, -0.54]	0.37
**Baseline TNF-α**						
≤ 10 pg/ml	4/93	-0.33 [-0.82, 0.17]		—	—	
> 10 pg/ml	3/249	-0.85 [-1.51, -0.18]	0.22	—	—	—
**Baseline IL-6**						
≤ 5 pg/ml	—	—		3/79	-0.31 [-1.27, 0.65]	
> 5 pg/ml	—	—	—	3/249	-1.23 [-1.93, -0.54]	0.13
**ACEI/ARB**						
Not all used	1/14	-1.41 [-2.62, -0.19]		0/0	—	
All used	5/292	-0.62 [-1.17, -0.08]	0.25	5/292	-1.10 [-1.60, -0.60]	—
**β-blockers**						
Not all used	3/125	-0.42 [-0.94, 0.09]		2/111	-0.05 [-1.17, 1.07]	
All used	4/217	-0.71 [-1.34, -0.07]	0.50	4/217	-1.33 [-1.74, -0.92]	0.06
**EPA + DHA**						
≤ 1000 mg/d	3/133	-0.23 [-0.57, 0.11]		3/133	-0.26 [-1.06, 0.54]	
> 1000 mg/d	4/209	-1.01 [-1.50, -0.53]	0.01	3/195	-1.47 [-1.84, -1.11]	0.007
**EPA**						
≤ 800 mg/d	4/266	-0.51 [-1.20, 0.19]		4/266	-0.62 [-1.54, 0.29]	
> 800 mg/d	3/76	-0.76 [-1.25, -0.27]	0.56	2/62	-1.25 [-1.93, -0.56]	0.28
**DHA**						
≤ 1300 mg/d	4/266	-0.51 [-1.20, 0.19]		4/266	-0.62 [-1.54, 0.29]	
> 1300 mg/d	3/76	-0.76 [-1.25, -0.27]	0.56	2/62	-1.25 [-1.93, -0.56]	0.28
**EPA DHA ratio**						
≤ 1	3/210	-0.80 [-1.53, -0.07]		3/210	-0.88 [-2.20, 0.44]	
> 1	4/132	-0.38 [-0.79, 0.03]	0.32	3/118	-0.66 [-1.04, -0.29]	0.76
**Duration**						
≤ 4 months	5/168	-0.32 [-0.63, -0.01]		4/154	-0.38 [-1.02, 0.26]	
> 4 months	2/174	-1.15 [-1.67, -0.62]	0.008	2/174	-1.59 [-1.93, -1.25]	0.001
**Quality score**						
≤ 3	5/299	-0.77 [-1.32, -0.22]		4/285	-0.81 [-1.72, 0.10]	
> 3	2/43	-0.16 [-0.78, 0.46]	0.15	2/43	-0.80 [-1.45, -0.15]	0.99

p^※^ difference between strata (Student’s *t* test).

TNF-α, tumor necrosis factor α; IL-6, interleukin 6; CHF, chronic heart failure; NYHA, New York Heart Association; LVEF, left ventricular ejection fraction; ACEI, angiotensin-converting enzyme inhibitor; ARB, angiotensin receptor inhibitor; EPA, eicosapentaenoic acid; DHA, ducosahexenoic acid; SMD, standardized mean difference; CI, confidence interval.

### Effects of fish oil supplementation on IL-1

Only two trials [18,23] reported the effects of fish oil supplementation on IL-1 in CHF (Table 2), between which no significant heterogeneity was found (I^2^ =46.0%, p =0.173). The pooled results indicated that fish oil reduced the level of circulating IL-1 (SMD = -1.24, 95% CI -1.56 to -0.91, p < 0.001; Figure 3B) in patients with CHF.

### Effects of fish oil supplementation on IL-6

Five trials [17-19,22,23] including 6 study arms reported the effects of fish oil on IL-6 in CHF (Table 2). Significant heterogeneity was found among these study arms (I^2^ = 85.8%, p < 0.001). The pooled results indicated that fish oil significantly reduced the level of circulating IL-6 (SMD = -0.81, 95% CI -1.48 to -0.14, p = 0.02; Figure 3C) in patients with CHF. However, sensitivity analysis, excluding the two study arms in which IL-6 data were imputed from median and IQRs [22], suggested an insignificant effect of fish oil on IL-6 (SMD = -0.81, 95% CI -1.72 to 0.10, p = 0.08).

The results of meta-regression analysis include some predefined covariates indicated that follow-up duration was negatively related to effect size (regression coefficient = -0.016, 95% CI -0.004 to -0.028, p = 0.017), which may partially explain the heterogeneity of the effect (Figure 3C).

Subgroup analysis showed that the differences in total dose of fish oil and the follow-up duration might influence the effects of fish oil on IL-6. Specifically, fish oil supplementation with a dose over 1000 mg/day or for duration over 4 months seemed to relate to a greater reduction of circulating IL-6 (Table 3).

### Effects of fish oil supplementation on sICAM-1 and sVCAM-1

**Figure 4:**
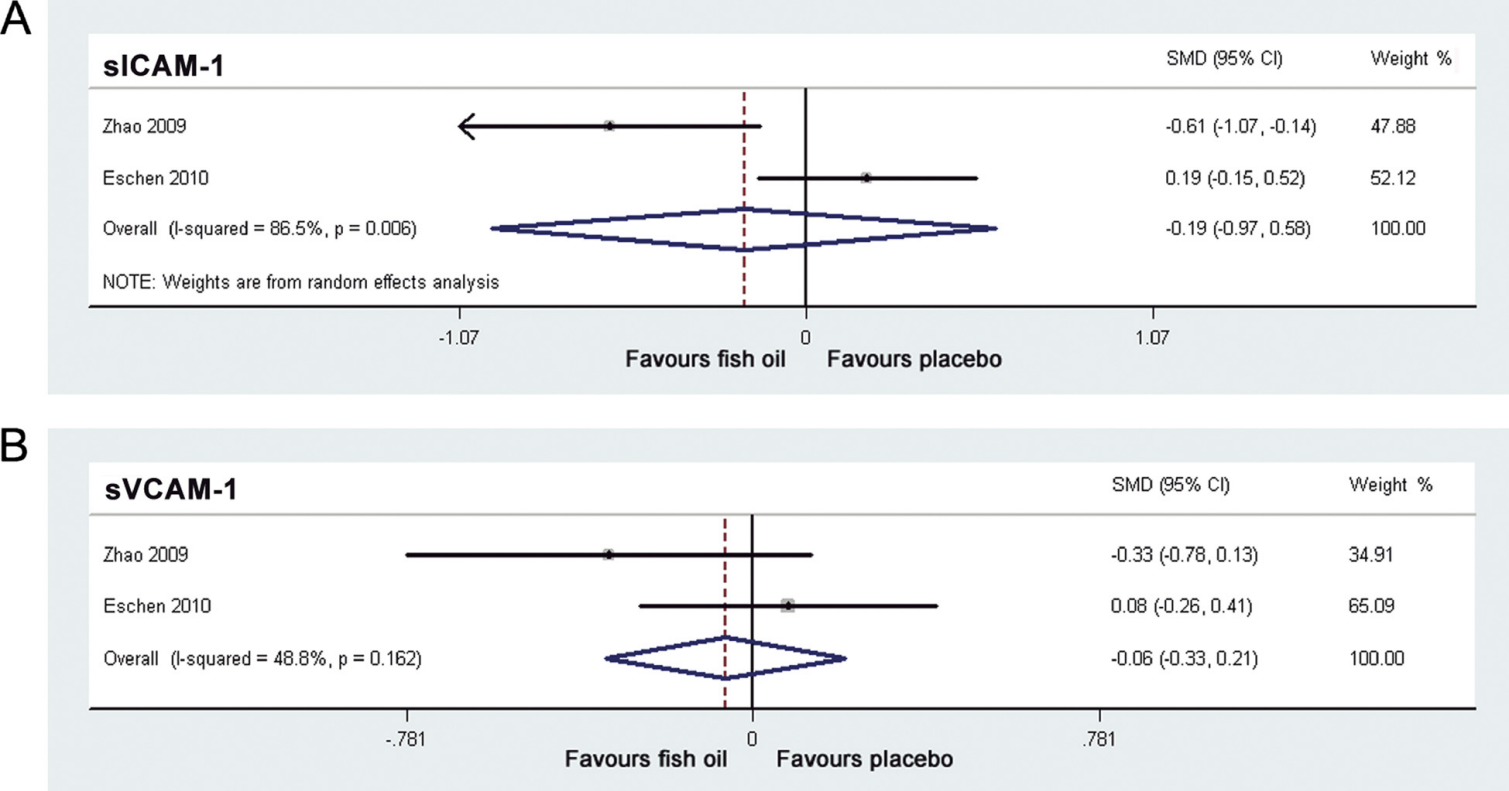
**Forest plots from meta-analysis of standardized mean difference in circulating soluble intracellular adhesion molecule-1 (A) and soluble vascular cell adhesion molecule-1 (B) for patients with CHF randomized to fish oil or placebo.** The effect size of each study is proportional to the statistical weight. The diamond indicates the overall summary estimate for the analysis; the width of the diamond represents the 95% CI. CHF, chronic heart failure; sICAM-1, soluble intracellular adhesion molecule-1; sVCAM-1, soluble vascular cell adhesion molecule-1; SMD, standardized mean difference; CI, confidence interval.

Only two studies [19,20] reported the effects of fish oil supplementation on sICAM-1 and sVCAM-1 (Table 2). For sICAM-1, estimation of the overall effects were performed with random-effect model because significant heterogeneity was found (I^2^ = 86.5%, p = 0.006); while for sVCAM-1, fixed-effect model was applied because no significant heterogeneity was found (I^2^ = 48.8%, p = 0.162). The pooled results indicated that additional supplementation of fish oil didn’t affect the levels of circulating sICAM-1 or sVCAM-1 (sICAM-1: SMD = -0.19, 95% CI -0.97 to 0.58, p = 0.63, Figure 4A; sVCAM-1: SMD = -0.06, 95% CI -0.33 to 0.21, p = 0.65, Figure 4B). Meta-regression and subgroup analysis was not performed for these outcomes because of the limited number of studies included. 

### Publication bias

**Figure 5:**
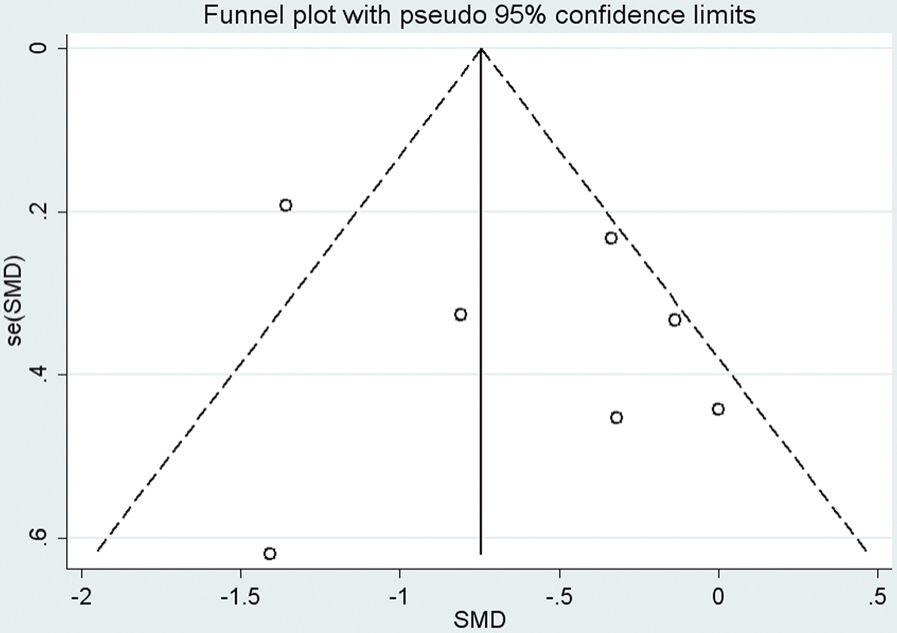
**Funnel plot (with pseudo 95% CIs) of all individual study arms in the meta-analysis of circulating TNF-α for patients with CHF randomized to fish oil or placebo.** CHF, chronic heart failure; TNF-α, tumor necrosis factor α; SMD, standardized mean difference; se, standard error; CI, confidence interval.

Publication bias was tested based on the data of TNF-α, which included the most study arms. Funnel plot (Figure 5) and Egger regression asymmetry test of the 7 included study arms suggested no significant publication bias for TNF-α (Egger test, p = 0.372).

## Discussion

In this meta-analysis, we summarized evidence from 7 published intervention trials that investigated the effects of fish oil supplementation on circulating markers of inflammation in patients with CHF. The results showed that fish oil reduced the circulating levels of TNF-α, IL-1 and IL-6 in patients with CHF, although the levels of hsCRP, sICAM-1 and sVCAM-1 were not significantly affected. Results of meta-regression and subgroup analysis suggested that supplementation with higher dose of fish or for a longer follow-up duration might associate with more remarkable reduction of TNF-α and IL-6 levels. These results suggested that anti-inflammation may be a mechanism underlying the potential benefits of fish oil for patients with CHF.

Results of a few epidemiologic studies demonstrated that fish consumption or fish oil intake was inversely associated with incidence of CHF in general population [30-32]. Furthermore, recently published GISSI-HF trial [9] showed that in patients with CHF on evidence-based therapy, long term treatment with fish oil reduced combined endpoint of mortality or hospitalizations for cardiovascular reasons. Besides, our recently published meta-analysis also indicated that fish oil supplementation can favorably affect the cardiac function and functional capacity in CHF patients [33]. However, the exact mechanisms of these beneficial effects of fish oil to patients with CHF are not fully understood [15].

Accumulating evidence from animal experiments and clinical investigations have revealed that CHF may at least partially be an inflammatory disease, where various cytokines and adhesion molecules are involved in CHF pathogenesis and progression via contribution to endothelial dysfunction, cardiomyocyte apoptosis, cardiac remodeling and left ventricular dysfunction [10,34-36]. These inflammatory markers, including hsCRP, TNF-α, IL-1, IL-6, ICAM-1 and sVCAM-1 et al., were found to be elevated in patients with CHF [37-42], and were associated with the severity of the disease and the prognosis of the patients [11-13]. Moreover, experimental evidence suggested that inflammation may also be a treatment target for CHF, and suppression of the above inflammatory factors or inhibition their adverse biological effects in CHF may be of great potential therapeutic significance [14,36].

It has been suggested that fish oil supplementation may exert anti-inflammation effects by decreasing the production of inflammatory cytokines and adhesion molecules in some of the inflammation associated diseases, such as rheumatoid arthritis, inflammatory bowels diseases and asthma, but there were also studies didn’t support this concept [43,44]. Furthermore, a recent systematic review in healthy subjects, subjects with cardiovascular risk and established cardiovascular disease indicated that intervention studies with fish oil in these population demonstrated rather different results on inflammatory markers, although the reasons for above controversy were unknown [45]. Since anti-inflammation was suggested to be involved in the therapeutic mechanisms of fish oil to cardiovascular disorders, we systematically evaluated the effects of additional fish oil supplementation on the circulating levels of inflammatory markers in CHF patients. The results of our meta-analysis indicated the suppressive effects of fish oil on circulating TNF-α, IL-1 and IL-6, suggesting the potential role of fish oil supplementation in blunting the inflammatory response associated with CHF and modifying the outcomes of these patients. Previous studies indicated that the potential mechanisms of fish oil on these inflammatory markers may involve its ability to modulation the nuclear transcription factor (NF-ΚB) [46,47], which subsequently inhibited transcription of above inflammatory markers in CHF. Besides, recent evidence suggested that fish oil may transcriptionally up-regulate adiponectin, a potential anti-inflammatory factor, thereby suppressing above inflammatory cytokines [48,49].

Considering the significant heterogeneity that existed in the pooled analysis of TNF-α and IL-6, we performed meta-regression and predefined subgroup analysis to explore the potential sources. The results suggested that the dose and follow-up duration of fish oil supplementation might influence the effects of fish oil on circulating levels of TNF-α and IL-6 in patients with CHF, and a higher dose of fish oil or a longer follow-up duration seem to be associated with a more remarkable reduction of circulating levels of TNF-α and IL-6. Notably, for the studies included in meta-analysis of IL-1 [18,23], the doses of fish oil are over 1000 mg/day and the follow-up durations are 6 to 12 months, which made the pooled result a reduction of IL-1 by fish oil supplementation.

High sensitivity C reactive protein is considered to be of prognostic value in many cardiovascular diseases [50,51], including CHF [13]. However, the pooled results of our meta-analysis failed to demonstrate a significant effect of fish oil supplementation on circulating level of hsCRP in patients with CHF. These results are supposed to be robust, because none of the included studies in meta-analysis of hsCRP showed a significant influence of fish oil supplementation on circulating hsCRP levels, and the heterogeneity among these studies was very little. The nonsignificant efficacy of fish oil supplementation on circulating hsCRP levels in the included CHF patients of this meta-analysis may be explained by the following two reasons. First, the patients of the included studies were in stable clinical and hemodynamical conditions receiving treatment of the optimal medications (including neurohormonal inhibitors in all studies and statins in some studies) according to the current guidelines. Therefore, the mean baseline circulating levels of hsCRP for the participants of included studies were relatively low (1.3 to 5.6 μg/ml, Table 2) and additional supplementation of fish oil had little effect on it. Second, the low number of included studies (4 studies) and the small size of the included population (285 patients) may make the analysis underpowered to show significant effect.

Our meta-analysis also failed to show a significant efficacy of fish oil supplementation on the levels of sICAM-1 and sVCAM-1 in CHF. These results, from our point of view, may also be attributed to the low number of included studies (2 studies) and the small size of the included population (213 patients).

Several potential limitations should be addressed regarding the present meta-analysis. First, the numbers of studies and patients included in this meta-analysis were small, so results of some of estimations, such as for the effects of fish oil supplementation on IL-1, sICAM-1 and sVCAM-1, should be interpreted with caution. Second, because baseline data of statins using in half of the included study arms were unavailable, we were unable to estimate the effects of statins using on the pooled analysis of the inflammatory markers. This may be an important source of heterogeneity. Third, as for the subgroup analysis, the number of included studies and patients in each stratum is relatively small. Besides, we did not have access to individual patient data. Some subgroup analysis of CHF patients might show a difference with fish oil supplementation if larger numbers of patients were included or individual patient data were available (e.g. fish oil supplementation on circulating IL-6 in ischemic and non-ischemic CHF patients). Fourth, this meta-analysis included one study [21,22] in which the data were imputed from median and IQRs for some outcomes. Although inclusion of the imputed data may add to the potential bias, sensitivity analysis by excluding this study showed generally consistent results with which all the studies were included. Furthermore, we considered this as the best possible approach to not exclude valuable data from related studies.

## Conclusion

In conclusion, our meta-analysis, by pooling the limited trials available currently, indicates that additional supplementation of fish oil may reduce the circulating levels of TNF-α, IL-1 and IL-6 in patients with CHF, although the levels of hsCRP, sICAM-1 and sVCAM-1 were not significantly affected. Also, greater reduction of TNF-α and IL-6 might be seen in patients who take fish oil in a higher dose or for a longer duration. These results suggested that anti-inflammation might be a possible mechanism underlying the potential beneficial effects of fish oil supplementation to patients with CHF. Additionally, large-scale randomized controlled trials with adequate power are warranted in the future to confirm these effects.

## Competing interests

The authors declare that they have no competing interests.

## Authors’ contributions

XL conceived of the study idea and XL and WX contributed to the study design. WX conducted the literature review. WX and WW performed the data extraction and all authors were involved in consensus agreements concerning data discrepancies. WX and XL drafted the manuscript. All authors were involved in revising the article for important intellectual content, interpreting the data, and approved the final version to be published.

## Pre-publication history

The pre-publication history for this paper can be accessed here:

http://www.biomedcentral.com/1471-2261/12/77/prepub
